# Early evaluation of the therapeutic effect of denosumab on tartrate-resistant acid phosphatase 5b expression in a giant cell tumor of bone: a case report

**DOI:** 10.1186/1756-0500-7-608

**Published:** 2014-09-05

**Authors:** Naoto Watanabe, Seiichi Matsumoto, Takashi Shimoji, Keisuke Ae, Taisuke Tanizawa, Tabu Gokita, Noriko Motoi, Teruko Ueno, Mitsuru Koizumi

**Affiliations:** Department of Orthopaedic Oncology, Cancer Institute Hospital, 3-8-31 Ariake, Koto-ku, Tokyo, 135-8550 Japan; Pathology, Cancer Institute Hospital, 3-8-31 Ariake, Koto-ku, Tokyo, 135-8550 Japan; Radiology, Cancer Institute Hospital, 3-8-31 Ariake, Koto-ku, Tokyo, 135-8550 Japan; Nuclear Medicine, Cancer Institute Hospital, 3-8-31 Ariake, Koto-ku, Tokyo, 135-8550 Japan

**Keywords:** Giant cell tumor of bone, Receptor activator of nuclear factor kappa-B ligand, Tartrate-resistant acid phosphatase 5b

## Abstract

**Background:**

Giant cell tumor of bone is an osteolytic, usually benign, tumor characterized by the infiltration of osteoclast-like giant cells. The receptor activator of nuclear factor kappa-B ligand pathway has been shown to play a key role in the pathogenesis of giant cell tumor. Treatment for refractory, recurrent, or metastatic giant cell tumor remains challenging. A monoclonal antibody to receptor activator of nuclear factor kappa-B ligand, denosumab, offers promise in these patients. Tartrate-resistant acid phosphatase 5b, a bone resorption marker, is secreted from osteoclasts and this marker is reported to be high in patients with giant cell tumor of bone. We investigated the effects of denosumab and the usefulness of a tartrate-resistant acid phosphatase 5b as a monitoring marker in the management of a refractory giant cell tumor of bone.

**Case presentation:**

A 41-year-old Japanese male with right ischiac pain was diagnosed with a giant cell tumor in his right ischium. Since the tumor extended to the acetabulum, there was a possibility that en bloc resection might significantly impair function of the hip joint and curettage could cause massive bleeding. Therefore, denosumab therapy (120 mg, administered 3 times every 4 weeks) was performed before radical surgery. The giant cell tumor of bone was treated with denosumab successfully. No adverse reaction was noted. Tartrate-resistant acid phosphatase 5b secretion was measured in the patient’s serum to monitor the response to denosumab, and a rapid normalization of the marker was observed after the first denosumab administration.

**Conclusion:**

This case suggests that denosumab therapy might be an option for treating refractory giant cell tumor of bone, and that tartrate-resistant acid phosphatase 5b might be an early marker with which to monitor the efficacy of denosumab therapy for refractory giant cell tumor.

## Background

Giant cell tumor (GCT) of bone is the most common, usually benign, bone tumor afflicting younger patients. Standard procedure for therapy is surgical resection. However, treatment options for unresectable cases have remained fairly static, and consequently treatment for refractory, recurrent, or metastatic GCT remains challenging [1, 2]. The receptor activator of nuclear factor kappa-B ligand (RANKL) pathway has been shown to play a key role in the pathogenesis of GCT [3, 4]. A monoclonal antibody to RANKL, denosumab, offers promise in these patients [5]. Tartrate-resistant acid phosphatase (TRACP) 5b is secreted from osteoclasts and is reported to be elevated in the serum of patients with GCT of bone [6]. We investigated the efficacy of denosumab and evaluated the usefulness of TRACP 5b as a marker to monitor the management of a refractory GCT of bone.

## Case presentation

**Figure 1:**
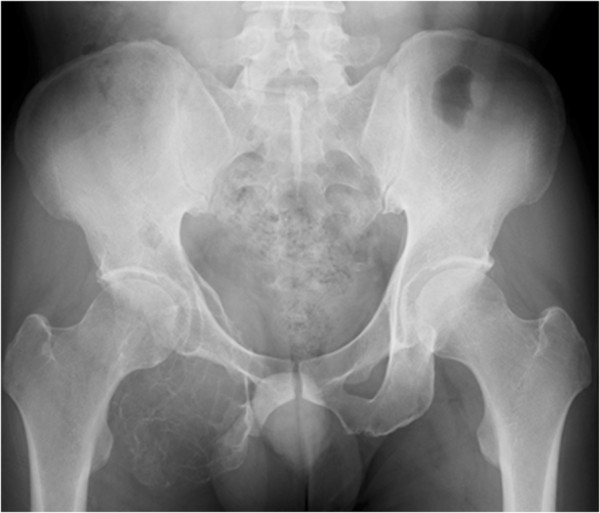
**X-ray showed a tumor of the right ischium with thinning of the cortical bone.**

**Figure 2:**
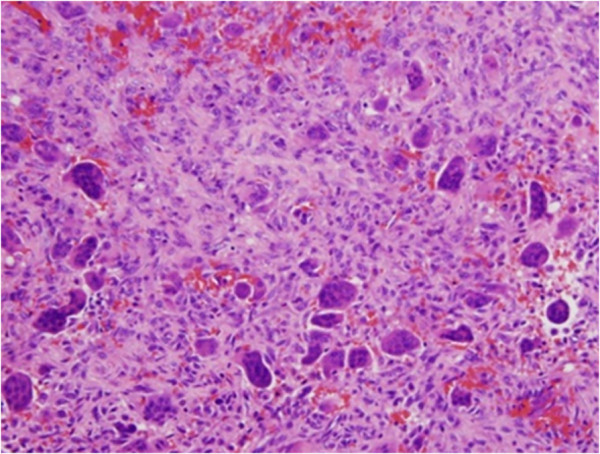
**Incision biopsy was performed (intraoperative bleeding: 170 mL).** Histology showed interstitial mononuclear cells lacking atypical features and multinucleated giant cells. The patient was diagnosed with GCT.

**Figure 3:**
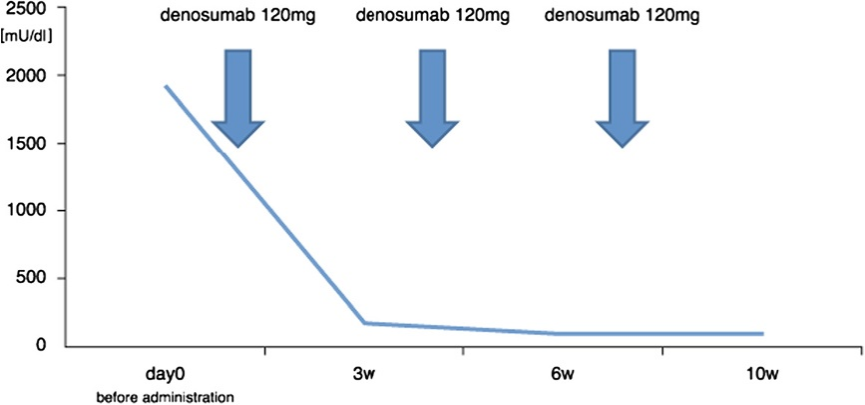
**TRACP 5b expression changes after administration of denosumab.** After the first administration of denosumab, TRACP 5b returned to within the normal range.

**Figure 4:**
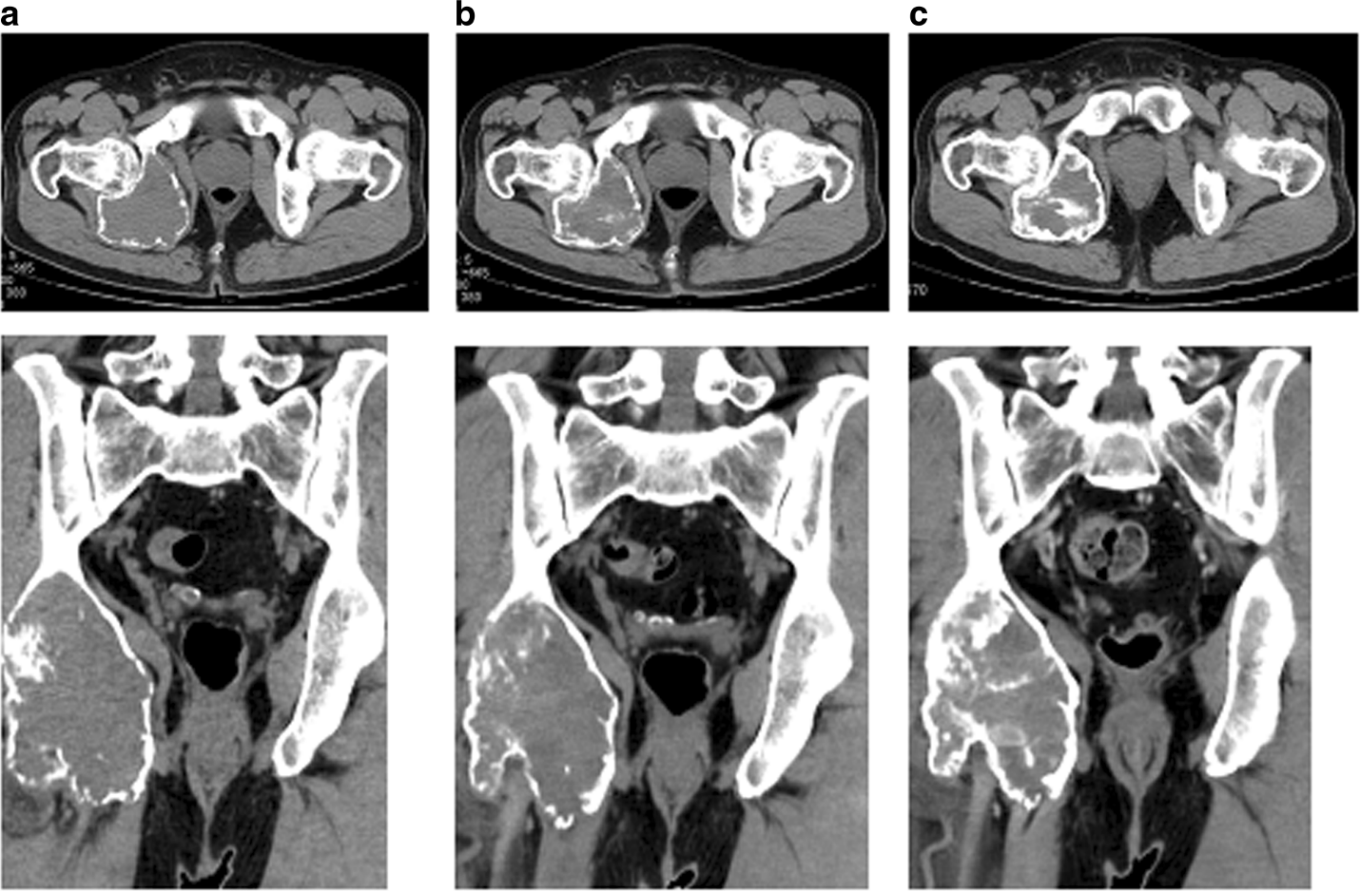
**Analysis of pelvic CT images before administration (a), 1 month after denosumab administration (b) and 3 months after administration (c).** Shell formation and cortex remodeling were observed at the tumor margin following denosumab administration.

**Figure 5:**
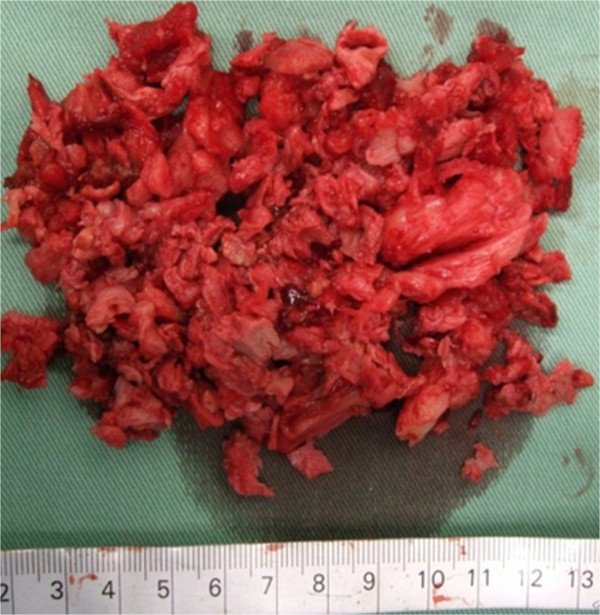
**Macroscopic view of surgical samples after denosumab therapy.** The lesion visibly contained both scar-like tissues and bone tissues, without evidence of any tumor.

**Figure 6:**
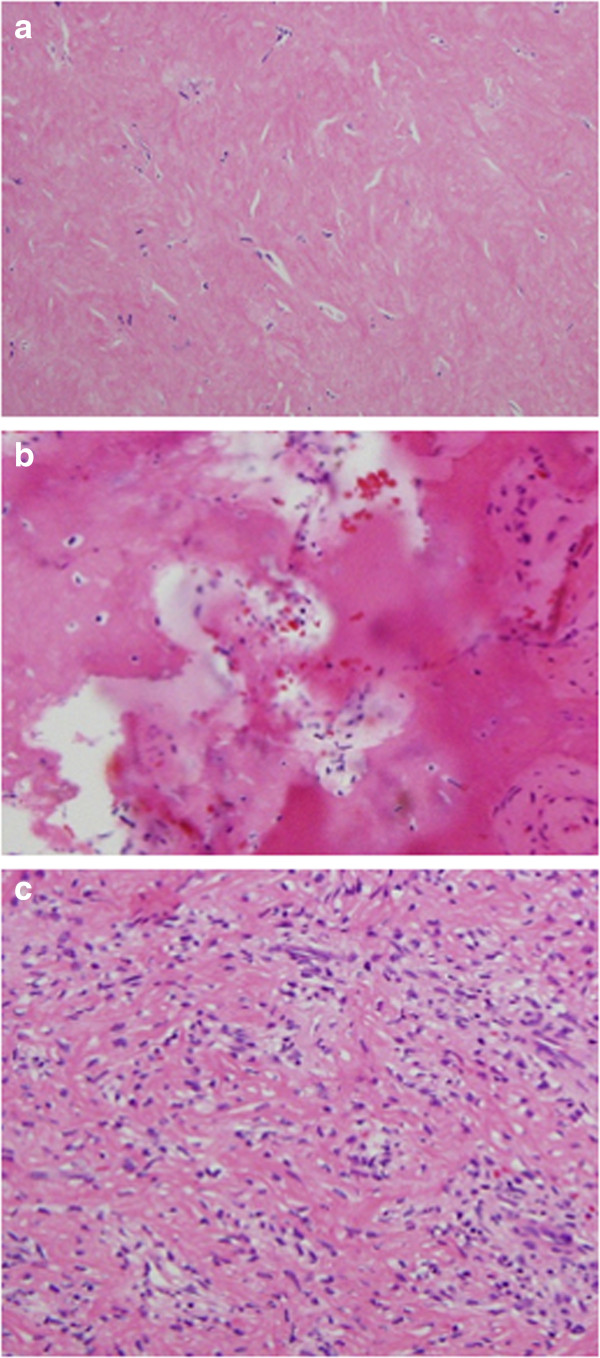
**Tissue specimen obtained after the administration of denosumab. (a)** The tumor cells completely disappeared and were mostly replaced by fibrous cells. **(b)** Tissues showed evidence of partial bone formation. **(c)** Specimens had high cell densities but contained only aggregated inflammatory cells.

A Japanese 41-year-old man with type II diabetes mellitus visited a nearby hospital with a major complaint of right ischiac pain that had persisted for 1 year. Plain X-ray revealed an osteolytic bone tumor with thinning of cortex in the right ischium (Figure 1). He was referred to our department for examination and treatment. At his first visit, he presented with spontaneous pain and tenderness in the right ischium. Blood test showed high TRACP-5b levels (1920 mU/dl; normal value: ≤590 mU/dl). The results of the blood tests showed no other abnormal findings. Magnetic resonance imaging (MRI) showed a low signal intensity area on both T1- and T2-weighted images, and the tumor exhibited contrast enhancement with gadolinium.Based on the X-ray and MRI findings, GCT of the ischium was suspected. Incision biopsy was performed (intraoperative bleeding: 170 mL), and histopathological findings showed interstitial mononuclear cells lacking atypical features and the presence of multinucleated giant cells. Thus, a diagnosis of GCT was established (Figure 2). The tumor extended to the acetabulum, and therefore it was possible that en bloc resection might significantly impair the function of the hip joint. Additionally, the level of curettage required could cause massive bleeding. Therefore, a non-surgical approach was first employed, using denosumab (120 mg) as an adjuvant therapy thrice weekly, every 4 weeks.After the first dose of denosumab, TRACP-5b levels rapidly decreased to normal values (181 mU/dl), and remained within the normal range (Figure 3). Additionally, with continued denosumab treatment, we observed shell formation and cortex remodeling at the tumor margin by serial computed tomography (CT) examinations (Figure 4).Angiography and embolization were performed 35 days after the third course of denosumab therapy and, on the following day, surgical treatments, including tumor curettage and artificial bone (hydroxyapatite) grafting, were carried out. Intraoperative bleeding was 1700 ml. Curetted tissues visibly contained only bone tissues and fibrous tissues, and no tumorous tissues with suspected GCT could be found (Figure 5). Pathological examination also showed that the multiple GCTs detected before surgery had disappeared and were replaced by fibrous cells. No residual GCT was found in the specimen, indicating the effectiveness of the denosumab therapy. The tissues showed partial reactive bone formation, which was considered to be bone regeneration, and the presence of aggregated inflammatory cells (Figure 6).

Based on the modern interpretation of pathophysiology, GCT of bone is now regarded as a predominantly osteoclastogenic stromal tumor [1]. Numerous cells stain positively for both RANKL and stromal cell factor-1 [7]. The GCT stromal cells are now widely understood to be the major neoplastic and proliferative component of GCT [1]. These stromal cells recruit monocytes by secreting various chemokines [1], with monocytes shown to express RANK and stromal cells, RANKL [4]. This RANKL pathway is essential for osteoclast maturation. The results of a Phase 2 study have indicated that denosumab offers disease and symptom control for advanced or refractory disease [5]. Denosumab interferes with this RANKL pathway and was indeed effective against the growing osteoclast in the present case. The question still remains whether stromal cells can be eliminated completely by prohibiting the RANKL pathway. Fortunately, no GCT could be found in the pathological examination of this case after denosumab therapy. Among bone resorption markers, TRACP 5b is the only marker secreted by osteoclasts [8], and has been reported to increase in patients with GCT of bone [6]. In our case, TRACP 5b dramatically decreased after the first denosumab administration, and serial measurements of marker showed that its concentration in the blood reflected the pathological GCT activity. TRACP 5b may thus be a good marker to monitor the GCT activity in patients undergoing treatment with denosumab.

## Conclusion

The results of this case study suggest that denosumab might be a potential therapeutic option for refractory GCT of bone, and that TRACP 5b could be used as an early marker to monitor denosumab therapy for patients with refractory GCT.

## Consent

Written informed consent was obtained from the patient for publication of this Case Report and any accompanying images. A copy of the written consent is available for review by the Editor-in-Chief of this journal.

## Abbreviations

GCT: Giant cell tumor
RANKL: receptor activator of nuclear factor kappa-B ligand
TRACP: Tartrate-resistant acid phosphatase
MRI: Magnetic resonance imaging
CT: Computed tomography.
